# 

**Published:** 1853-01

**Authors:** 


### CONTENTS.

### ORIGINAL COMMUNICATIONS.

Page.
Dr. Drake's Interrogatories, ... 59
Filling Teeth. By Dr. Clark, ... 79
Springing of Plates. By Dr. J. Harris, ... 83
Extraction of Teeth. By Dr. J. Taylor, ... 84
Dentists Fees. By Dr. C. P. Culver, of New Castle, Ky., ... 91
Dr. Morton vs. Dr. Jackson—Sulphuric Ether, ... 94
Filling Teeth over Exposed Nerves. By S P. Miller, of Worcester, Mass., ... 107

### EDITORIAL.

Risodontrypy, or Hullihen's Operation, ... 113
Prof. Harris' Principles and Practice of Dental Surgery, ... 113
The Southern Journal of the Medical and Physical Sciences, ... 114
New Dental Works, ... 114
Dental Expositor, ... 114
Porcelain Teeth, ... 114
Dr. B. A. Satterthwaite,—Lima, O., ... 114
World's Fair at New York, ... 115
Illustrated Magazine of Art, ... 116
Dr. J. Harris on Springing of Plates, ... 116
Mississippi Valley Association of Dental Surgeons, ... 116
The Ohio Dental College Association, ... 116
Drs. Allen and Hunter,—Priority of Improvement, ... 116
Prize Essays, ... 117
Prize Essays ... 117
Dental Furnishing Establishments, ... 117
Death of Professor Drake, ... 118
Death of Drs. Stipp and Cleaveland, ... 118

